# Determination of Drugs in Clinical Trials: Current Status and Outlook

**DOI:** 10.3390/s22041592

**Published:** 2022-02-18

**Authors:** Babak Tavana, Aicheng Chen

**Affiliations:** Electrochemical Technology Centre, Department of Chemistry, University of Guelph, 50 Stone Road East, Guelph, ON N1G 2W1, Canada; btavana@uoguelph.ca

**Keywords:** clinical trials, analytical methods, drugs, sensors and biosensors, MEMS

## Abstract

All pharmaceutical drugs, vaccines, cosmetic products, and many medical breakthroughs must first be approved through clinical research and trials before advancing to standard practice or entering the marketplace. Clinical trials are sets of tests that are required to determine the safety and efficacy of pharmaceutical compounds, drugs, and treatments. There is one pre-phase and four main clinical phase requirements that every drug must pass to obtain final approval. Analytical techniques play a unique role in clinical trials for measuring the concentrations of pharmaceutical compounds in biological matrices and monitoring the conditions of patients (or volunteers) during various clinical phases. This review focuses on recent analytical methods that are employed to determine the concentrations of drugs and medications in biological matrices, including whole blood, plasma, urine, and breast milk. Four primary analytical techniques (extraction, spectroscopy, chromatography, and electrochemical) are discussed, and their advantages and limitations are assessed. Subsequent to a survey of evidence and results, it is clear that microelectromechanical system (MEMS) based electrochemical sensor and biosensor technologies exhibit several notable advantages over other analytical methods, and their future prospects are discussed.

## 1. Introduction

### 1.1. Clinical Trials

Clinical trials are research studies that serve to improve quality of life through the evaluation and monitoring of new drugs and medical procedures. All drugs, medications, and medical tools are required to pass all phases of clinical trials (one pre-phase and four phases) to meet governmental requirements and be allowed to transition from laboratory R&D to the marketplace. Researchers assess clinical results to evaluate the appropriate dosages and efficacy of emerging drugs, and determine the safety, reliability, and advantages of new medical tools. The comparison of medical expectations against actual data from test results assists them with optimizing risk/benefit ratios, and further developing or re-designing new drugs and medical devices/systems. Each clinical human research trial includes criteria such as age, gender, and health condition that guide the selection of patients (or volunteers) who are invited to participate in a given study.

**Scheme 1:**
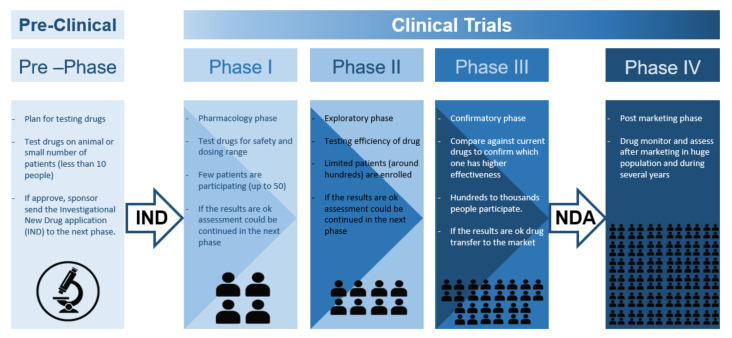
Illustration of Clinical Trial phases.

Generally, there is one pre-phase and four individual phases in a clinical trial. Initial pre-clinical phase trails are not mandatory; however, most pharmaceutical or medical companies perform them to decide which drug exhibits the best pharmacokinetics parameters in humans (or animals). This phase is performed with animals or a small number of patients (up to 10 people). If the results confirm expected effects, the next phase (Phase I) may ensue. Phase I (pharmacology phase) is the smallest phase in clinical trials, wherein researchers determine the pharmacokinetic and pharmacodynamic parameters of new drug candidates. Only a few patients participate in this phase (up to 50), as researchers aim to identify safe dosages (how much of the drug is safe) and treatment solutions (how to optimize treatments for maximum effectiveness), while continuously evaluating the vital signs of patients and monitoring them for side effects. If the dosage safety and efficacy is approved, the new treatment can continue to the next phase (Phase II). In Phase II (Exploratory phase), researchers investigate the safety and efficacy of a drug or treatment; they need to elucidate how drugs affect the human body and determine exactly how the treatment works. A limited number of patients (hundreds) are enrolled for this phase. If the results are approved, the advantages and disadvantages of the new method are assessed, and the drugs are ready to pass to the next phase (Phase III). In Phase III (Confirmatory phase), many participants are required (hundreds to thousands of individuals). Here, new drugs or treatments are compared against currently available treatments to confirm which one best meets the established criteria. If these results are acceptable, manufacturers may register a New Drug Application (NDA) and transfer it to the market. In Phase IV (post marketing), the newly released drugs are assessed and monitored after marketing to a significant population over several years to evaluate any side effects over the long term. Scheme 1 illustrates the various clinical trial phases.

### 1.2. Drug Classifications

**Scheme 2:**
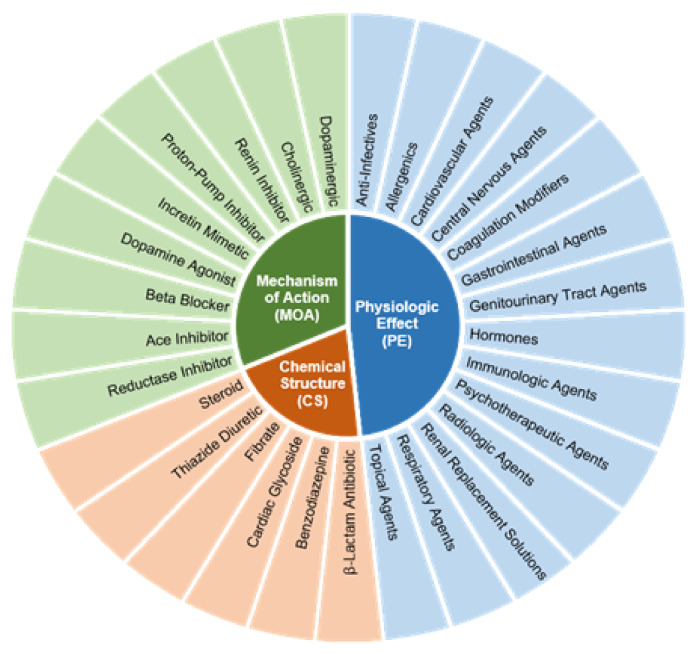
Illustration of pharmacological classifications and examples of the three attributes.

There are two main classifications for drugs: therapeutic and pharmacological. The therapeutic classification describes why a drug is used or expresses any physiological changes that are induced by the drug in the body. However, the pharmacological classification explains the drug’s mechanism of action at the molecular level. Pharmacological interactions between drugs and biomolecules such as proteins (enzymes or receptors) are assessed. Therefore, this classification is more specific and is primarily used to assess drugs, rather than therapeutic classifications [1]. As per FDA guidelines [2], pharmacologic classes are defined based on the following three drug attributes: Mechanism of Action (MOA), Physiologic Effect (PE), and Chemical Structure (CS). Scheme 2 depicts pharmacological classifications, three drug attributes, and a few examples of each.

## 2. Analytical Methods

The development and validation of an analytical method in clinical research and bioanalysis for the determination of pharmaceutical compounds and drugs is a very complex and challenging process that includes several unexpected issues and is a very time-consuming procedure. To meet established guidelines and regulatory requirements, researchers need to formulate regulations that support development, validation, and data analysis. They need to extract the main analyte across biological samples, while monitoring and measuring it by one of the analytical techniques to be described below. Each method possesses its own advantages and disadvantages; thus, selecting one of them is contingent on the type of drug, methodological conditions, and limitations. In the following section we focus on four core analytical methods in clinical trials, encompassing extraction, spectroscopic, chromatographic, and electrochemical methods. Although techniques such as extraction have been integrated with other quantitative techniques such as chromatography or electrochemistry to provide higher precision and accuracy, they are described separately here to illustrate their evolution over the two decades and to compare their strengths and weaknesses.

### 2.1. Extraction Methods

Extraction is a technique that involves the separation of a specific portion of a mixture from other components. All extraction methods explain how a desired analyte is transferred from the first phase into the extracting phase. In fact, all solvable materials are dissolved in the solvent, after which any other insolvable residues are separated. This process depends on mass transfer where the main feature of the extraction rate is diffusion at the boundary interface. Historically, extraction has been employed for the preparation or separation of samples (qualitative analysis). However, recently, and particularly for clinical research, quantitative analysis and precise quantities of compounds in mixtures are required. Consequently, new extraction methods have been designed or coupled with other quantitative techniques such as chromatography or electrochemistry to provide higher precision and more accurate results. In clinical research, depending on which method is suitable and exhibits the best performance for the extraction of analytes, extraction methods are selected. The four most common types of extraction techniques are liquid/liquid, liquid/solid, chemical activity, and supercritical fluid extraction [3,4,5].

#### 2.1.1. Solid/Liquid Extraction (SLE)

For this extraction method, the desired analyte is in the solid phase, which is dissolved and mixed with the liquid (solvent) phase. Thus, the compound is transformed from the solid to the liquid phase and separated by filtration. For some techniques, in addition to filtration (depending on the analyte) additional steps such as drying water (or solvent), preconcentration, ultrasound, and agitation are required to complete the extraction process. In current clinical and pharmaceutical research, there are three main types of solid phase extraction: maceration, percolation, and supercritical fluid extraction. In this section, we focus on the supercritical fluid extraction (SFE) [6].

SFE is a major type of solid/liquid extraction that employs supercritical fluids as the extraction solvent. The main matrix is typically solid; however, sometimes this method is used for liquid matrices as well for the aforementioned reason; thus, it is categorized as the SLE type. One of the most-used extracting agents for SFE is carbon dioxide (CO_2_), which is a supercritical solvent with a critical temperature of 31 °C and critical pressure of 74 bars. These conditions are known to be fully compatible with pharmaceutical compounds and biological matrices. These substances might have temperatures and pressures higher than their critical ratio. The main properties of a supercritical fluid are its capacity to diffuse through solids and dissolve primary analytes. The SFE mechanism includes a heating container that the supercritical fluid (CO_2_) is pumped into as a liquid to attain the supercritical condition. Subsequently, the CO_2_ is introduced to the solid sample to dissolve the analyte. The dissolved compounds are transferred to a separate container with lower temperature and pressure conditions for settling [5].

Hofstetter et al. used SFE for the quantitative determination of a ketamine metabolites in biological matrices [7], whereas Baldino et al. reported the same extraction technique for the extraction of cannabis and other pharmaceutical compounds [8]. There are a few advantages for this method, including the absence of any solvent residue. Moreover, CO_2_ is non-toxic; non-flammable; and can be cooled, recycled, and reused. Furthermore, the supercritical conditions used for this technique, such as temperature and pressure, are flexible, which can be modified for different analytes. Conversely, this method is expensive and difficult for maintenance and operation and has high power requirements [9].

#### 2.1.2. Liquid/Liquid Extraction

Liquid/liquid extraction (LLE) transfers desired analytes from one solvent to another, with the extraction ratio being dependant on the properties of the solvents (solubility) involved. In fact, for analysis, the second solvent must have higher solubility than the first solvent to be able to dissolve within itself, while separating it from the first phase. The two liquid phases must be immiscible, mostly polar and non-polar, such as aqueous and organic based solvents. The LLE is specifically applied for the separation of organic compounds in pharmaceuticals. There are several mechanisms involved in LLE, some of which are important and useful, particularly for clinical research into ion exchange and ion pair extraction [10,11]. Although there are several extraction mechanisms for LLE, we focus only on ion pair extraction (IPE), stabilize the analytes, optimize the chromatogram shape, and improve the retention time [12].

Lan et al. used IPE and combined this method with HPLC tandem mass (MS/MS) to determine rosuvastatin in human plasma and improve the technique [13]. In another experiment, Studzińska et al. coupled IPE with mass spectrophotometry to determine the concentration of oligonucleotides in living cells [14]. Panganiban et al. used this approach for the quantitative determination of cocaine and its metabolite in a biological matrix. In these experiments, researchers coupled IPE with the chromatography method and used IPE in the structure of the chromatography column to improve extraction.

#### 2.1.3. Solid Phase Extraction (SPE)

**Figure 1:**
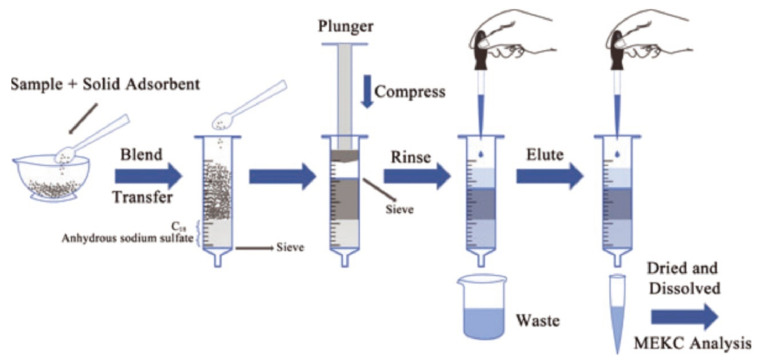
Four steps of SPE: conditioning, loading, washing, and elution. Reprinted by permission from [15]. Copyright 2018 Elsevier.

For this technique, the desired compounds (liquid or gas) are dissolved in the solvent (mobile phase) and interacted with a solid phase based on their chemical and physical properties. In the next step, the analyte is dissolved and rinsed out with another solvent, after which its concentration is determined. Generally, there are four steps involved in SPE that include conditioning, loading, washing, and elution, as illustrated in Figure 1 [15]. In the first step (conditioning) an extraction cartridge must be prepared and be free from impurities. For the second step (loading) the analyte stock solution is loaded into the cartridge. The third step (washing) involves rinsing for the removal of any adsorbed interferents from the SPE cartridge. In the last step (elution) the analyte is rinsed out with a proper solvent. There are a few types of SPE, including reversed phase, normal phase, and ion exchange. Reverse phase and ion exchange will be discussed in the chromatography section [16].

In the most recent research, SPE has been reported to be combined with other characterization techniques (chromatography and biosensors) to improve its accuracy and selectivity. Wang et al. employed SPME (solid phase microextraction) in the structures of chromatography columns and coupled it with HPLC for the determination of anti-inflammatory drugs [17]. Mpupa et al. also applied an MIP-SPE combination for the analysis of hormones in biological matrices [18].

### 2.2. Spectroscopic Methods

Spectroscopy utilizes the detailed knowledge of radiative energy beams and materials. Using this method, the structures of materials and the concentration of analytes can be analyzed through an evaluation of the interactions between the radiative beam and the target material. There are five primary spectroscopic techniques, including Raman, Ultraviolet-Visible (UV/Vis), Nuclear Magnetic Resonance (NMR), Infrared (IR), and X-Ray. The fundamental principle between all these techniques is to shine a beam onto the sample and evaluate the response of the sample to this stimulus. In this section, we endeavor to review some of the current and new spectroscopic methods that are employed for the determination of drug concentrations for clinical research [19].

#### 2.2.1. Ultraviolet-Visible (UV/Vis)

For the ultraviolet-visible (UV/Vis) method, the sample is placed in front of a light source. When the light has passed through the sample, the molecules therein absorb energy (light), and their electrons are transferred and promoted from low energy level orbitals to higher state energy level orbitals. The discrete wavelengths of UV or visible light that are adsorbed (or transmitted) by sample are compared with the wavelengths of a blank [20]. In clinical research, Scheeren et al. compared two determination methods (reverse phase HPLC versus UV-Vis spectrophotometry) for the determination of doxorubicin [21]. UV-Vis is typically combined with the other instruments (e.g., chromatography method, HPLC-UV) as a detector to determine pharmaceutical compounds and drugs. Bulduk utilized reverse phase HPLC combined with a UV detector for the determination of favipiravir [22].

This technique does not damage the analyte following analysis; thus, the sample can be reused. Further, this method is rapid and inexpensive, and the instrument is simple to operate. Conversely, this method requires sample preparation if there are multiple absorbing species or solid particles in the solution. Additionally, several other features such as pH, contamination, temperature, and impurities might have negative effects on the results. Finally, this method can only be used for liquid samples; therefore, gasses or solids cannot be analyzed via this technique [22,23,24].

#### 2.2.2. Raman Spectroscopy

Raman Spectroscopy is a subset of vibration spectroscopy that provides information regarding the chemical structures, identities, phases, molecular interactions, contamination, and impurities of analytes based on their vibrational characteristics. Akin to several spectroscopic methods, a laser beam (from high intensity laser source) is emitted into the sample, after which the detector identifies the scattered light that returns [19]. As per Rayleigh scattering, most of the detected scattered light has the identical wavelength as the original laser beam, which is not useful. However, some of the light that is scattered at different wavelengths is called Raman scatter. This scattering is dependent on the chemical structure and molecular interaction of the sample. Each molecular bond, such as C-C, N-O, or C-H, possesses a different vibration and thus has a different peak position in the Raman spectrum. The resulted spectrum is unique and can be used to identify samples or analyze their detailed chemical behaviours. Further, by employing the intensity of the peaks and finding a relation between the concentration and intensity, the concentration of the sample is calculable (quantitative measurement) [25].

Because Raman scattering signals are weak, surface-enhanced Raman scattering (SERS) has been employed in clinical trials for the detection of drugs with acceptable sensitivity and selectivity. In SERS, a plasmonic metal is used to adsorb target molecules, which can increase the Raman scattering signal. The biological matrices used with this method may be whole blood, plasma (serum), urine, saliva, or tears. SERS has also been employed in clinical research to diagnose different types of cancer (e.g., prostate, breast, and colon), as well as to identify diseases such as eye glaucoma [25].

**Figure 2:**
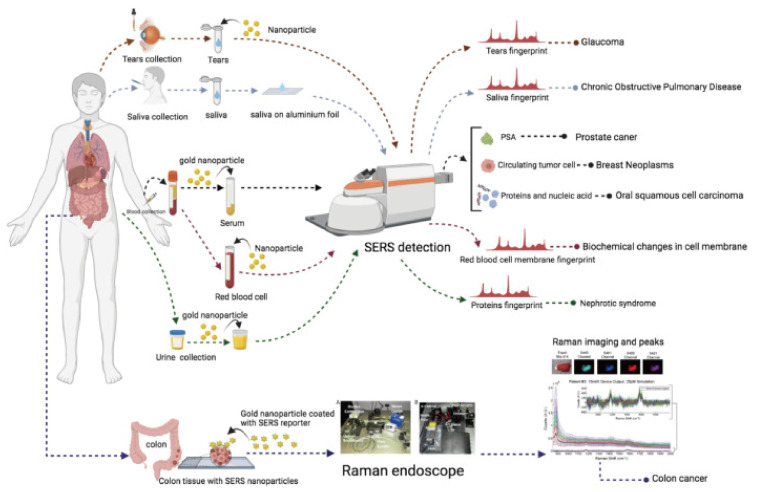
Application of surface-enhanced Raman scattering spectroscopy (SERS) in clinical trials. Reprinted by permission from [28].

Frosch et al. monitored pharmaceutical drugs in clinical research using Raman spectroscopy [26]. Additionally, Tanwar et al. utilized advanced Raman spectroscopy techniques to analyze materials and characterize pharmaceuticals [27]. Xi et al. evaluated the application of surface-enhanced Raman scattering spectroscopy (SERS) in clinical trials, as illustrated in Figure 2 [28]. This method is suitable for a number of organic and inorganic mixtures; thus, it can support an extensive range of pharmaceutical compounds. No sample preparation is required, and the analysis is quick. Conversely, this technique cannot be used for metals; some sample characteristics such as fluorescence might alter the spectrum or shift it, and certain samples may be destroyed by the laser beam [29].

#### 2.2.3. Fluorescence Spectroscopy

**Figure 3:**
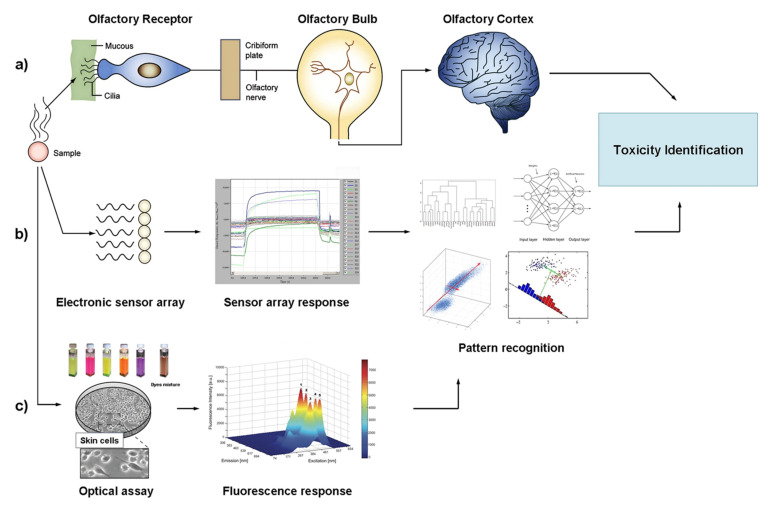
Fluorescence-based assay as a new screening tool for toxic chemicals in clinical trials, (**a**) human olfactory system, (**b**) electronic analogue, and (**c**) our optical analogue. Reprinted by permission from [35].

For fluorescence spectroscopy, analyte residing electrons are excited using a photonic beam (typically UV), after which the light emitted from the analyte is measured by a detector. Generally, the mechanism of action for this technique is fluorescence resonance, which is the transition of electrons from the first level excited state to the ground state [30]. The measurement devices for fluorescence are quite like those for other spectrophotometers. The beam emitted from the light source passes through filter fluorometers and reaches the sample. This filter lets only a portion of the light pass, which excites fluorescence, whereas the remaining wavelengths are eliminated. The emitted beam reaches the detector after passing through a second filter, after which the beam is amplified by a detector and displayed on an oscillator [31,32]. Qin et al. used fluorescence spectroscopy to determine carcinoembryonic antigens in clinical research [33], whereas in another experiment Xu et al. combined an electrochemical method based on molecular imprinted polymer (MIP) to detect caffeic acid and used fluorescence spectroscopy as a detector [34]. Moczko et al. developed a diagnostic assay based on the fluorescence spectroscopic method to monitor toxic chemicals in biological matrices, as illustrated in Figure 3 [35].

Fluorescence spectroscopy exhibits high sensitivity and specificity due to its optical properties with a large range of linearity. Additionally, this technique is superior to similar spectroscopic methods in some respects due to the low background and immunity to light scattering. However, the major weakness of this method is that only fluorescent samples can be analyzed. An additional disadvantage of this technique is that fluorophore lifespans are short [30,31,32].

#### 2.2.4. Infrared (IR) and Fourier Transform Infrared (FTIR) Spectroscopy

IR spectroscopy is a powerful tool for the detection and monitoring of pharmaceutical compounds and drugs in clinical environments and biological matrices. This technique is a subset of molecular vibrational spectroscopy, which can determine the structures of molecules and mechanisms of chemical reactions according to their molecular absorption. When an IR beam hits the sample, some of this light is absorbed and some is transmitted. The IR spectra are the results of molecular absorption and transmission, which are referred to as a molecular fingerprint. Because each fingerprint is unique and different from other fingerprints, each IR spectrum may be different from others and represents individual components (molecular structures). In fact, each spectrum includes adsorbed peaks that reveal the atomic bonds of the analyte, where the intensity of the peaks indicates the analyte quantity (concentration) [36].

The radiation absorbed by molecules alters the dipole moments of the sample molecules, where consequently, electrons are transferred from the ground to excited states [37]. By analyzing the IR spectrum, abundance data regarding the structures of molecules may be easily obtained. For example, vibrational energy can be evaluated from the frequency of the peaks, as the number of the vibrational molecules are related to the number of the peaks. Furthermore, the transition of vibrational states (energy levels) is directly related to the intensity of the absorbed peaks. The principal of FTIR is the same as IR, where the only difference between the FTIR and IR is the interferometer that is used in the FTIR configuration. The generated beams pass through an interferometer, which splits them into two different paths. Because of the difference between the optical path length, the intensity and retardation of each beam are different. Finally, the signals are amplified and converted via an amplifier and converter, respectively, and the two beams are recombined and detected by detector [38].

**Figure 4:**
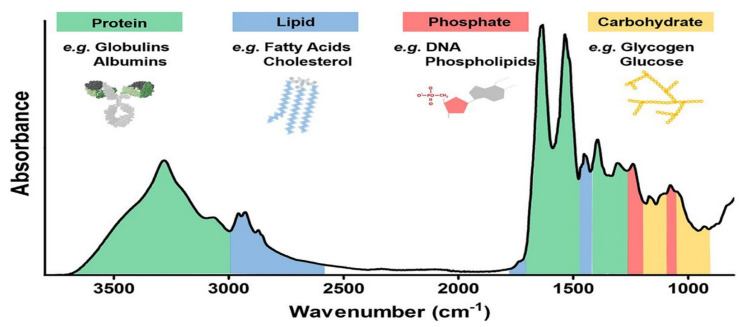
FTIR spectroscopy for biomolecules in human blood. Reprinted by permission from [41]. Copyright 2020 Elsevier.

Essousi et al. used FTIR to characterize the MIP used in an electrochemical sensor for the determination of pharmaceutical samples [39]. In other clinical research carried out by Nosrati et al., FTIR results were used to confirm the components and functionalization of magnetic albumin nanoparticles [40]. Sala et al. employed FTIR spectroscopy to diagnose cancer in the early stage with high sensitivity and specificity in pre-clinical trials, as illustrated in Figure 4 [41]. FTIR spectroscopy has several advantages, such as high sensitivity, as well as rapid and inexpensive analysis that can cover all types of analytes (gas, solids, and liquids). Conversely, the major limitation of this technique is that it cannot analyze molecules that are not active in the IR region [42].

#### 2.2.5. Nuclear Magnetic Resonance (NMR) Spectroscopy

NMR spectroscopy can determine the electronic structures and chemical properties of molecules. When analytes are surrounded by a magnetic field, the energy states of atomic nuclei change, and an NMR signal is produced. All nuclei have spin and electrical charge, and due to the radio frequency wavelength (external magnetic field) the energy level is transferred from the base state to higher levels. When spin returns to the original position (base state), energy is emitted. The signal corresponding to this energy is measured, and its output is processed as the NMR spectrum [43].

**Figure 5:**
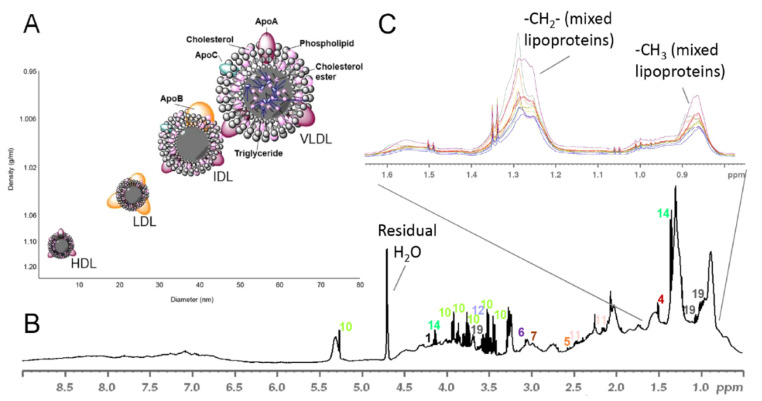
(**A**) Density and size of lipoprotein composition (**B**) 1H NMR spectrum of a serum sample. (**C**) Different lipoprotein signals from several samples with differing compositions. Reprinted by permission from [44]. Copyright 2018 ACS Publications.

Jiménez et al. used NMR spectroscopy to determine lipoproteins and their metabolites in human plasma and serum, as illustrated in Figure 5 [44]. De Castro et al. reviewed metabolomics studies (chemical processes including metabolites, small molecule substrates, intermediates, and the products of cell metabolism) and the application of NMR in metabolite determination in clinical drug research [45].

This method can provide detailed chemical structures without destruction (non-destructive method). In addition, it is an inexpensive solution that requires minimal maintenance. On the other hand, there are some limitations, including low sensitivity and the requirement of a large quantity of sample or a long analysis time for some methods, such as C NMR, H NMR, and TD-NMR, and only qualitative results may be provided due to the overlapping of signal to noise for TD-NMR [45].

### 2.3. Chromatographic Methods

Chromatography is one of the most important separation techniques that is used to detect and qualitatively or quantitatively measure pharmaceutical drugs and chemical components. Analysis can be distinguished based on molecular structure, functional group, charge, and interactions with stationary phases. Generally, there are four main types of separation techniques (ion exchange, adsorption, partition, and size exclusion) that operate based on interactions between target molecules and a stationary phase [46]. For these methods, the mobile phase assists with moving a mixture of molecules throughout the column. Interactions between the stationary phase and the mixture separate target analytes based on adsorption (liquid-solid), partition (liquid-solid), or the difference in molecular weight. Therefore, some portions of the mixture have stronger and more stable interactions with the stationary phase than the others and move slower in the chromatography column. Other portions move faster with the help of the mobile phase, and they appear and are identified by the detector [47,48,49].

#### 2.3.1. Continuous Column Chromatography (CC)

**Figure 6:**
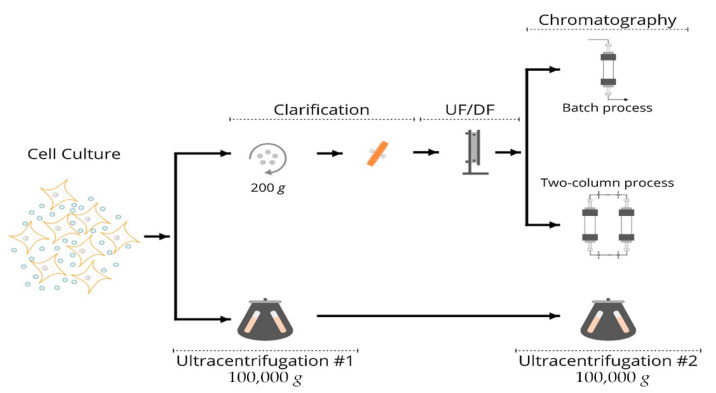
Workflow of the EV purification process. Purification method based on clarification (low-speed centrifugation and filtration), ultrafiltration, and size exclusion chromatography, which can be operated in batch or continuous mode (top route); isolation method based on two ultracentrifugation stages of 100,000× *g.* Reprinted by permission from [50]. Copyright 2019 Elsevier.

For the purification of extracellular nanovesicles, continuous column chromatography (two-columns system) has been designed for the separation of extracellular vesicles (EVs) from cellular supernatants. Products are segregated based on their size and vesicle formation, as shown in Figure 6 [50]. The multi-columns method (continuous purification) can be run for large-scale products. In this technique, the first (XK16/20) and second columns (XK16/70) are packed with a specific exclusion resin (Sepharose 4 Fast Flow 17014901, GE Healthcare, UK) with an average size of 90 µm and tested as per manufacturer’s procedure. The exosome dosage was from 0.1 to 200 µ [50]. This method is a reliable automated process that has high potential for most complex mixtures; however, it is time-consuming and expensive (as it requires higher quantities of the mobile phase) with low separation power.

#### 2.3.2. Ion-Exchange Chromatography (IEC)

IEC is one of the most popular types of chromatography that separates compounds based on the charges of chemicals and biological groups. In clinical research it is primarily used to purify and separate proteins, enzymes, peptides, amino acids, and antibodies. Matsuda et al. [51] reported an application of IEC with UV and mass spectrometry detectors for analyzing the sites of antibody–drug conjugates (ADCs). ADC is a biopharmaceutical drug that is used for the treatment of cancers, and analyzing them using IEC is commonly employed. Due to the presence of nonpolar groups in the structures of ADCs, a combination of reverse phase liquid chromatography and mass spectrometry detectors are typically used. This method is one of the most effective and powerful techniques for separating charged particles, which can cover all charged molecules. Although the cost of columns is low due to the long life of resins, it is an expensive technique because the costs involved in the preparation of buffers are high.

#### 2.3.3. Molecular Sieve or Gel-Permeation Chromatography (GPC)

Molecular Sieve or GPC is a type of liquid chromatography that is used to separate biomolecules such as proteins, polysaccharides, enzymes, and polymers in clinical trials. For this chromatographic technique, columns are packed with a stationary phase that is comprised of matrices such as polystyrene, dextran, polyacrylamide gels, or agarose gels. Stationary phase gels function like molecular sieves that can separate analytes based on their molecular size. Chen et al. [52] used high performance gel chromatography coupled with a charged aerosol detector (HPGPC-CAD) for qualitatively analysis and ultraperformance liquid chromatography combined with tandem mass detectors (UPLC-QqQ-MS/MS) for the quantitative method validation of the determination of polysaccharides in herbal medicine. In this clinical study, HPGPC was used to determine the molecular weight of polysaccharides using tandem GPC columns. The combination of two columns and a CAD detector enhanced the chromatographic resolution and provided a more reliable molecular weight-retention time calibration curve. Simplicity and reliability are the two main advantages of this method; however, inadequate equipment with low-capacity sample volumes and separation is its main disadvantages.

#### 2.3.4. Affinity Chromatography (AC)

AC is one of the most popular separation methods for the analysis of enzymes and proteins in pharmaceutical and clinical studies. It is also used in genetic engineering, biological compound purification, and vaccine production. For this method, separation is accomplished based on a reversible interaction between analytes and specific ligands such as antibodies and antigens, enzymes and substrates, or enzyme and inhibitor interactions [53,54]. Cao and Liu [55] employed high performance affinity chromatography (HPAC) to determine the binding relations between the structures of plasma proteins and human serum albumin (HAS). A CHIRAL-HAS column that was specifically designed for HAS was used, revealing that different structures of the main analyte and changing molecular functional groups had different protein binding rates.

The AC method has high sensitivity and specificity for analysis and purification of compounds and can be utilized by pharmaceutical manufacturers to evaluate the quality of products (vaccines). This technique is reliable, with a high degree of purity that can also be used in genetic engineering. Conversely, the maintenance and operation of the instrumentation involved is complicated. There is also a limitation in the volumes of sample and ligands, which make it an expensive method.

#### 2.3.5. Paper Chromatography (PC)

PC is a technique for the characterization of molecules with low masses based on their distribution in mobile and stationary phases. Because this method is not suitable for large populations of biomolecules, complex mixtures, or volatile products, with resulting precision and accuracy that is not adequate for clinical trials, most researchers prefer to use other chromatography methods, such as HPLC, AC, or HPTLC, and continue the development of other methods to enhance quantitative analysis [56].

#### 2.3.6. Thin-Layer Chromatography (TLC)

**Figure 7:**
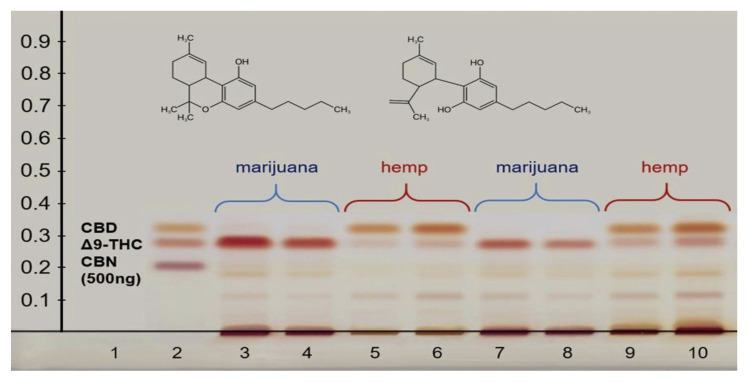
High performance thin-layer chromatography (HPTLC) analysis of cannabinoids. Reprinted by permission from [58]. Copyright 2020 Elsevier.

TLC is a type of affinity chromatography, with its stationary phase being a layer of adsorbent compounds such as silica gel or aluminum oxide on a glass or alloy surface. The mobile phase moves up through the plate, where sample components also transit based on capillary forces with the mobile phase but with different elution speeds, as per interactions between the sample component and stationary phase. When the mobile phase reaches the end, the plate is dried to find the spots and calculate the retention time [57]. In some clinical trials, the lipophilicity of a drug is an important parameter that can reflect its functionality in the body; thus, HPTLC methods with a silica gel plate as a stationary phase have been widely used for the determination of pharmaceutical compounds, as illustrated in Figure 7 [58]. Although this is a simple, cheap, and user-friendly technique, there are some limitations in cases of volatile compounds or a high number of samples. Further, the data and results are not as accurate as other chromatographic methods such as HPLC and GC.

#### 2.3.7. Gas Chromatography (GC)

GC is a popular technique for the separation of vaporized pharmaceutical compounds. The overall concept is the same as liquid chromatography; however, the difference is that for this method the sample is vaporized immediately following injection. For vaporization, the temperature of the oven chamber is set higher than the boiling points of the samples. The vaporized analyte is carried via a mobile phase directly to the column (stationary phase). The mobile phase in this technique is a type of non-reactive gas, such as He, which has no interaction with the analyte; thus, separation is based on the adsorption ratio between the analyte and the stationary phase. Chaumont et al. [59] used this method to analyze the effects of e-cigarettes on lung inflammation. In another clinical study, exhaled breath was analyzed using two dimensional GC coupled with a mass spectrometry detector [60]. Generally, GC is a powerful method that can separate a wide range of volatile analytes with high resolution and sensitivity. GC analysis is also fast in contrast to other chromatography techniques and has an excellent signal to noise ratio. However, the main weakness of this separation method is that only volatile and thermally stable analytes may be injected and analyzed. Additionally, the selectivity of liquid chromatography is higher than GC due to the wide range of mobile phases.

#### 2.3.8. Dye-Ligand Chromatography (DLC)

**Figure 8:**
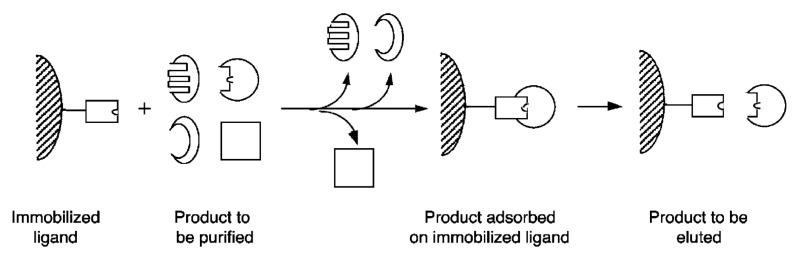
Principle of dye-ligand chromatography. Reprinted by permission from [62]. Copyright 2001 Elsevier.

DLC is one of the main types of affinity chromatography, which is primarily used to purify a wide variety of molecules such as proteins, enzymes, coenzymes, cofactors, antibodies, and amino acids in biological matrices [61]. The configuration of the instrument is almost the same as for AC techniques; however, the column in these methods is packed with dye ligands that can interact with the active sites of proteins, and these bonds can be either hydrophobic, hydrogen, or electrostatic. The affinity interactions between ligands and analytes are affected by pH, temperature, and particle dimensions, as illustrated in Figure 8 [62]. This is an inexpensive and user-friendly technique that is compatible with large sized analytes; however, there are limitations in using this method, as dye-ligands are not as selective and specific as other types of ligands.

#### 2.3.9. Hydrophobic Interaction Chromatography (HIC)

Hydrophobicity is an important parameter for the selection and separation of analytes. For the HIC separation method, molecules are separated based on relationships between water and hydrophones. Technically, biohydrophobics are non-polar molecules with long carbon chains that repel water, and the surfaces of these molecules have different degrees of hydrophobicity. In separation, molecules interact with the hydrophobic ligand in the chromatography column (which is packed with resins) with a high concentration of buffer. As soon as the concentration of the buffer changes, the degree of interactivity between the molecules and ligand is altered. Therefore, molecules with weak hydrophobicity wash out first, whereas molecules with a high degree of hydrophobicity that possess stronger bonds remain longer. Bobaly and Fleury-Souverain [63] separated Antibody Drug Conjugates (ADCs) using this technique and reported the results for a clinical study. They showed that using mild conditions such as physiological pH, temperature of mobile phase, with no organic solvent, not only helped to protect the structure of target molecules (e.g., proteins) but is also one of the main advantages of the HIC technique over other chromatography methods [64].

#### 2.3.10. High-Performance Liquid Chromatography (HPLC)

HPLC is one of the most commonly used chromatography techniques in clinical trials, where the main difference between this method and other traditional techniques (e.g., LC) is that HPLC relies on special pumps to pressurize the mobile phase and sample mixture through the column, leading to a shorter operation time and higher performance. Because most studied biomolecules and pharmaceutical compounds are hydrophobic, the dominant configuration for chromatography used in clinical studies is reversed-phase high-performance liquid chromatography (RP-HPLC), which is based on hydrophobic binding between main analyte and ligands in a non-polar stationary phase. In the structure of RP columns, the stationary phase is comprised of non-polar hydrocarbons that are bonded to silica [65]. The length of hydrocarbon depends on the separation parameters such as the size of molecules, hydrophobicity, retention time, density, etc.

For example, column C8 contains Octylsilane (OH-Si-C8) and has a short carbon chain, low hydrophobicity and density, short retention time, and fast sample elution, and is suitable for small organic compounds; however, column C18 contains octadecylsilane (OH-Si-C18) and has a longer carbon chain, higher hydrophobicity and density, a longer retention time, and slower sample elution, and is suitable for heavier and longer chain organic samples [66]. RP-LC and RP-HPLC can be coupled with UV or mass spectrometric detectors and applied for the determination of many commercial medications and pharmaceutical drugs. HPLC is a fast, reproducible and efficient technique with high accuracy and precision in contrast to other chromatography techniques. Conversely, it is expensive, with complex maintenance and preparation procedures. Table 1 ranks the most-used chromatographic methods based on application, advantages, and disadvantages.

### 2.4. Electrochemical Methods

The main disadvantages of the aforementioned analytical methods include time-consuming sample preparation, costly materials, and complex troubleshooting and instrumentation maintenance. The advantages of electrochemical methods (e.g., electrochemical sensors and biosensors) are that they can not only cover all these deficiencies, but also improve the level of analysis and the determination of pharmaceutical compounds. High sensitivity, high selectivity, a wide concentration range, low cost, rapid analysis, and a requirement for small quantities of analyte make electrochemical sensors and biosensors an attractive approach for the analysis of pharmaceutical compounds and drugs in clinical trials. Based on the drugs and pharmaceutical compounds that need to be analyzed, electrochemical methods are distinct. Relevant molecules such as proteins, small molecules, nucleic acids, enzymes, antibodies, and biomarkers encompass most categories of drugs and bio-compounds that can be determined using these techniques [67]. There are five main electrochemical methods for the determination of drugs in clinical trials, including voltammetry, potentiometry, conductometry, amperometry, and impedimetric.

#### 2.4.1. Sensors and Biosensors Based on Potentiometry

Potentiometric sensors and biosensors involve a static electrochemical method, where the difference in potential between a working and reference electrode is measured in an electrochemical cell. Most potentiometric methods are employed in the preparation of wearable sensors for monitoring and evaluating the vital signs of patients and volunteers in clinical trials Phases I to III [68,69]. Further, this method can be used to design sensors and biosensors for the determination of biomarkers or drugs in biological matrices [70,71]. Light-addressable potentiometric biosensors, MIP-based potentiometric biosensors, and potentiometric photoelectrochemical biomarkers are examples of applications of this method in clinical trials [72]. Brainina et al. used potentiometric sensors to evaluate the activities of dermal antioxidants [73].

A designed sensor may include three main components: a reference electrode, an indicator electrode, and a membrane. For example, a carbon screen printed electrode (CSPE) was used as an indicator and modified with gold nanoparticles. The modified electrode was defined as CSPE/AuNPs and used as an indicator electrode. The reference portion of the screen-printed electrode (SPE) was modified with silver and silver chloride. The final component of the sensor was the membrane that served as the carrier of the mediator, which helps to transfer electrons from the active side of the antioxidant to the working electrode and includes phosphate buffered saline [73]. This method is simple and cheap with high efficiency; but its main disadvantage is the instability of the potential, with temperature fluctuations that might shift the potential [74].

#### 2.4.2. Sensors and Biosensors Based on Voltammetry and Amperometry Methods

Both amperometry and voltammetry are dynamic electrochemical methods and are subclasses of controlled potential techniques. The only difference between these two methods is the type of exerted potential. In amperometry and voltammetry, fixed and variable potentials have been used, respectively. In three electrode systems, the potential with respect to the reference electrode is exerted on the working electrode, where the current flow between the working and auxiliary electrodes is measured as a function of induced potential. The most commonly used working electrodes include gold, silver, platinum, or carbon, whereas the reference electrode is comprised of saturated Ag/AgCl or a saturated calomel electrode (SCE), and the auxiliary electrode is made of platinum wire. Nitrogen or argon gas is used to remove dissolved oxygen in the electrolyte solution. The most common forms of voltammetry techniques include DC polarography, linear sweep, pulse, square wave, stripping, differential pulse, cyclic, and hydrodynamic [75,76].

##### DC Polarography Voltammetry (PV)

DC PV is an old and basic physicochemical method that uses a dropping mercury electrode (DME). By creating potential between a detector (polarisable electrode) and a reference electrode (non-polarisable), the potential can be measured, with the resulting potential referred to as a polarogram. The working electrode (DME) is a capillary glass tube, wherein mercury drops with a constant ratio from the tip of the electrode due to gravity. These droplets are continuously produced, and this function prevents contamination of the electrodes and stabilizes the condition of analysis [76]. Although polarography is one of the traditional electrochemical methods, it is not useful in clinical research due to some of the following limitations. The first and most disadvantage is its limited potential range; mercury can be oxidized at a potential of more than +0.2 V; thus, many analytes cannot be analyzed. The low sensitivity, difficulty in data interpretation, and the toxic nature of mercury are the other limitations [77].

##### Linear Sweep Voltammetry (LSV)

In three electrode systems, the current is measured while the potential applied to the working electrode is swept linearly over time. Oxidation or reduction occurs at the working electrode [78]. Hui et al. reported using the LSV method for the quantification of nanoparticles in biological matrices [79], whereas Filik and Avan coupled solid phase extraction with an LSV method for the separation and extraction analysis of chromium ions [80].

##### Pulse Voltammetry (PV)

To increase the speed and sensitivity of voltametric techniques, oscillations in potential are developed for some methods [76]. Two of these techniques are more commonly used than others: square wave voltammetry (SWV) and differential pulse voltammetry (DPV].

##### Square Wave Voltammetry (SWV)

**Figure 9:**
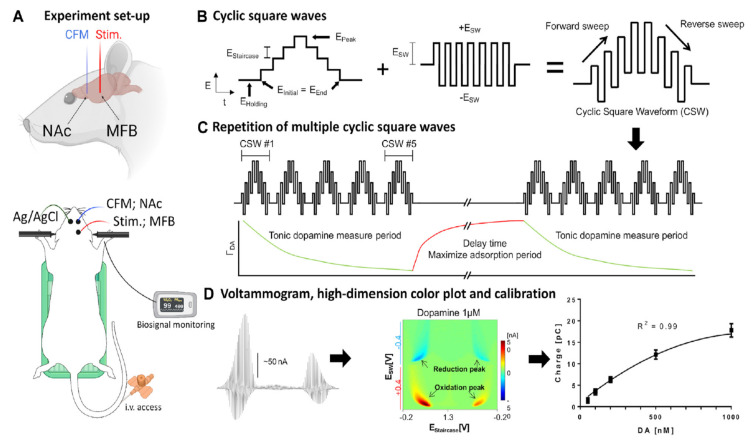
Setup of clinical voltammetry research. (**A**) Rat surgery setup. Recording and stimulating electrodes were inserted unilaterally into the core of the nucleus acumens and medial forebrain bundle, respectively. The counter-reference Ag/AgCl electrode was inserted contralaterally into cortical tissue. The rat was placed in a stereotactic frame with tail vein access, heating pad, and a pulse oximetry monitoring electrode. Elements of the Figure were created using Biorender.com. (**B**,**C**) Schematic design of waveform-CSWV applied to the CFM and its response. (**D**) Left-to-right: raw voltammogram following the removal of background currents, high-dimensional pseudo-color plot, M-CSWV signal calibration with tonic dopamine experiment (*n* = 4 electrodes). Reprinted by permission from [83].

SWV is one of the most recent and useful methods for the determination of drugs in clinical research, and it consists of a symmetrical square-wave pulse that is superimposed on the staircase waveform of steps [81], resulting in an increase of its sensitivity. Zheng et al. prepared nano porous gold electrode and designed a DNA biosensor based on square wave voltammetry for the detection of lung resistance related protein gene [82]. In another clinical research study, dopamine concentration was measured using SWV, as illustrated in Figure 9 [83]. SWV exhibits much higher sensitivity than other voltametric methods due to the removal of the background current. On the other hand, method complexity and difficult data extraction are the weaknesses of this technique [84].

##### Differential Pulse Voltammetry (DPV)

**Figure 10:**
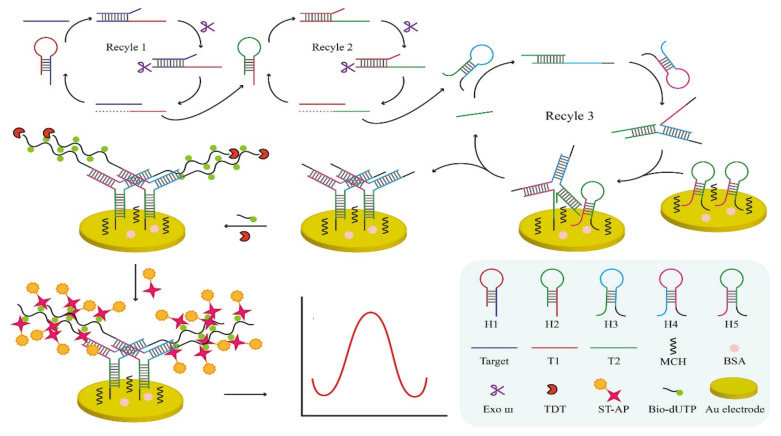
Working mechanism of electrochemical biosensor for the determination of DNA and amplified electrochemical signal quantified by DPV. Reprinted with permission from [87]. Copyright 2019 Elsevier.

The DPV technique is one of the most popular and most used voltammetry methods in clinical trials. For this method, a potential is applied to fixed amplitude pulses and superimposed on a linear potential. Prior to changing potential, the current is measured from two points: firstly, before the application of the potential, and secondly at the end of the pulse. The difference between these two points is determined and considered as a potential for each pulse [84]. Teófilo et al. used the DPV method to screen ecstasy compounds in the body using a boron-doped diamond electrode (BDD) [85]. In other research, DPV combined with solid phase extraction was used to measure irinotecan in a plasma matrix [86]. Huang, et al. used a type of DPV with a triple application to increase sensitivity for the detection of DNA, as illustrated in Figure 10 [87]. In contrast to other methods, DPV is a fast and simple technique, but it sometimes exhibits a lower sensitivity than SWV [88].

##### Stripping Voltammetry (SV)

**Figure 11:**
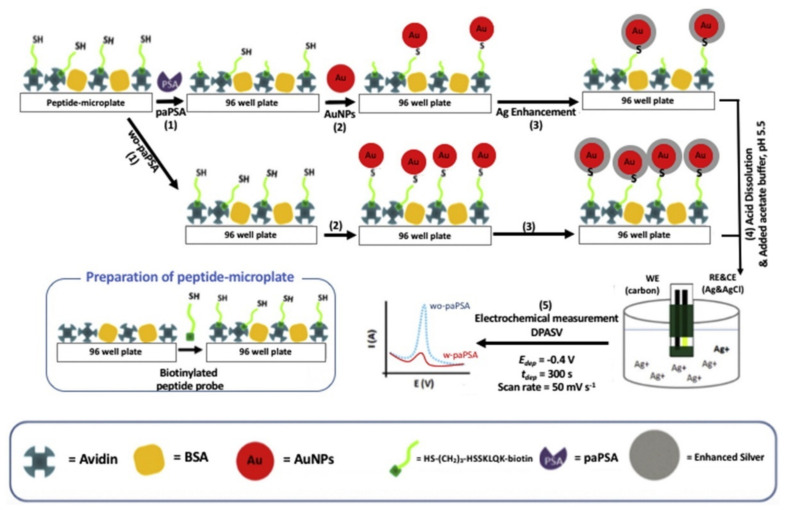
Determination of catalyzed peptide (paPSA) using differential pulse anodic stripping voltammetry (DPASV). Reprinted by permission from [92]. Copyright 2017 Elsevier.

SV is a powerful technique in electroanalysis that is based on voltammetry or potentiometry for the quantitative determination of ions in clinical research. The four main types of this method include adsorptive, anodic, cathodic, and potentiometric, which rely on preconcentration, deposition mechanisms, and stripping steps. For the detection of analytes, there are two steps. In the first step (accumulation step) the analyte concentration rises at the surface of the working electrode under an electrolysis condition (adsorptive, anodic, or cathodic), and in the second step (detection step) the reaction is exactly opposite to the first step, where the concentration of the analyte on the surface of the electrode decreases and strips the analyte from the electrode. Based on the first step condition, in the second step there are three possible stripping methods: adsorptive stripping voltammetry (AdSV), anodic stripping voltammetry (ASV), and cathodic stripping voltammetry (CSV) [89]. Because the sensitivity of the adsorptive methods is higher than the other methods and provides a lower detection limit, this method is widely used in clinical research. Ghoneim and Beltagi employed AdSV for the determination of celecoxib [90]. Jain et al. used the voltametric methods at hanging mercury drop electrodes (HMDE) to analyze and determine Cefixime in biological matrices [91]. In other research, differential pulse cathodic adsorptive stripping voltammetry (DPCAdSV) was used and compared with square-wave cathodic adsorptive stripping voltammetry (SWCAdSV) for the analysis and evaluation of antibiotic concentrations, as illustrated in Figure 11 [92]. This method is highly sensitive, inexpensive, and reproducible, and is comparable to many spectroscopic methods to determine pharmaceutical compounds, particularly metal ions. Conversely, stripping voltammetry has limitations for the detection of complex metals and also has environmental issues (waste) reported regarding mercury-coated electrodes [93].

##### Cyclic Voltammetry (CV)

**Figure 12:**
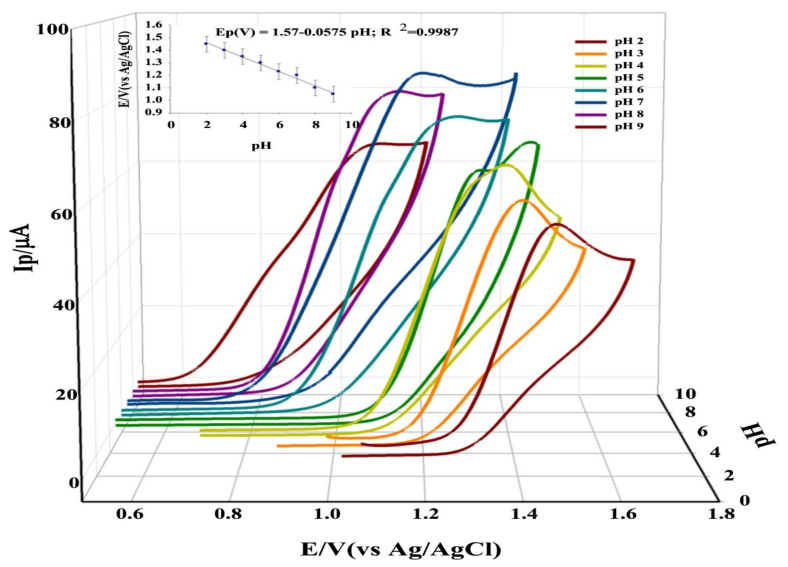
Cyclic voltametric responses of 1.0 mM COD at pH 2.0–9.0 using FSG/SPE sensing platforms at a scan rate of 0.1 V s^−1^. The inset displays the relation of IP versus pH. Reprinted by permission from [97]. Copyright 2018 Elsevier.

CV is one of the most extensively used electrochemical methods for qualitative analysis and potentiodynamic measurement. For the CV technique, the exerted potential is evaluated in both forward and reverse directions at the working electrode versus a reference electrode. When the potential of the working electrode in the forward direction reaches a set potential, the potential is ramped in the reverse direction back to its initial value [94]. Nikbakht and Pakbin used this method to evaluate a new method for lymphocyte reproduction [95]. In another experiment, fast scan cyclic voltammetry (FSCV) using hippocampal serotonin dynamics was determined in mice [96]. Mohamed et al. used a CV method as an initial electrochemical technique for the determination of codeine with modified screen-printed electrodes, as illustrated in Figure 12 [97]. This method might be employed for the determination of the mechanisms; analysis; characterization; and synthesis of pharmaceutical compounds, polymers, and metals. This method covers a wide variety of analytes and might be considered as a very reliable analysis method at the onset of all clinical research [98].

##### Hydrodynamic Voltammetry (HV)

**Figure 13:**
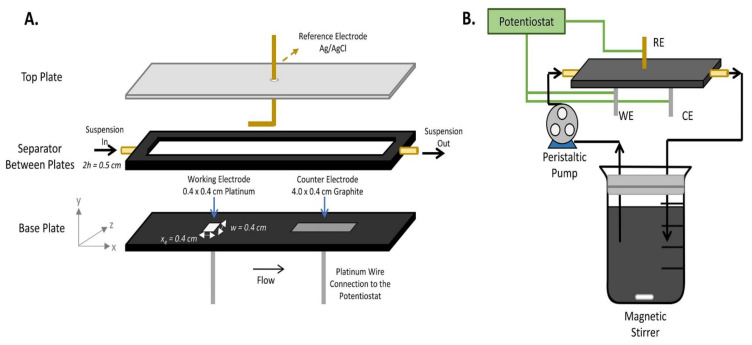
Hydro dynamic Cell. (**A**) Plates of Hydrodynamic Channel Cell (HCC) and (**B**) Electrochemical system. Reprinted by permission from [99].

Most electrochemical methods operate in a stagnant solution system. By introducing convection into the cell, the current and, therefore, sensitivity can be increased. The rate of the reaction at the surface of the working electrode can also be controlled by varying the convection rate in the solution. There are two possibilities for introducing convection into an electrochemical system. The working electrode can remain fixed, and the solution flow can be applied to the surface using an external force. Alternately, the structure of the electrode can be altered to mix the solution via convection. In this case, the flow of solution around the working electrode is laminar (smooth paths in layers), which provides a stable current. In HV, one can update the electrochemical system to a hydrodynamic setup and change the working electrode to a rotating disk electrode (RDE) and rotating ring-disk electrode (RRDE), as illustrated in Figure 13 [99]. Belli and Rossi used CV and HV with RRDEs to evaluate and monitor the reaction of antioxidants with superoxide radicals [100]. Bashir and Basharat used the HV technique to evaluate the rapid iodination of uracil and cytosine nucleobases in biological matrices [101].

#### 2.4.3. MEMS Sensors and Biosensors Based on Conductometry and Capacity

In this method, MEMS sensors and biosensors evaluate and measure the ratio of electrolytic conductivity changes in the sample to evaluate analyte (drug) concentration. It is the reciprocal of the more commonly encountered term, resistivity. Most molecules possess conductivity to different degrees, with some of them being low (e.g., benzene or silica) and some having a high degree of conductivity (e.g., silver, copper, and most metals). The determination mechanism is based on the measurement of resistance. This means that the sensor only requires two electrical contacts, where other electrochemical electrodes, such as reference electrodes, are not required. Perera et al. [102] designed a conductive sensor for the detection of biomarkers. Saiapina et al. [103] used conductometric sensors for the selective detection of ammonium solutions. As per experimental results, conductometric methods are fast, inexpensive, and repeatable; however, the main drawback is non-specific measurements. This means that the method cannot identify different types of ions; therefore, pre-knowledge prior to running the tests is essential [104,105,106].

#### 2.4.4. MEMS Sensors and Biosensors Based on Electrochemical Impedance Spectroscopy (EIS)

**Figure 14:**
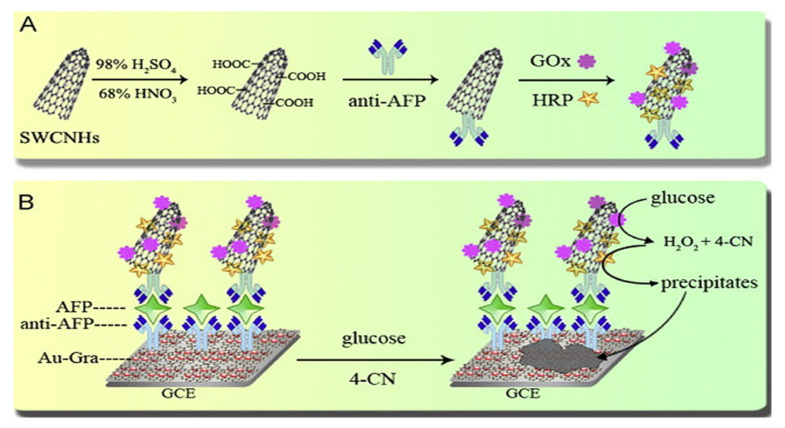
Preparation immunosensor (SWCNHs) based on electrochemical impedance spectroscopy (**A**) Stepwise functionality of a modified electrode (**B**), Modified glassy carbon electrode (GCE) by Au-Gra layer and anti-AFP antibody. Therefore, AFP will be detected in a sandwich configuration. Reprinted by permission from [109]. Copyright 2018 John Wiley and Sons.

EIS is a useful and efficient method for characterizing a wide range of electrochemical systems. For this method, a small amplitude alternating potential is applied to an electrochemical cell, and the system response to it is monitored. Due to the small amplitude of the excitation voltage, the EIS method is a non-destructive technique [107]. An EIS system includes an electrochemical cell, frequency generator, and frequency response analyzer (FRA), where the FRA is the heart of the system. In this system, analytes are used as working electrodes. Gazze et al. used a graphene biosensor based on the EIS method for the early diagnosis of ovarian cancer [108]. Figure 14 illustrates an immunosensor based on EIS measurements [109]. This method has a minimum amount of destructivity among all other electrochemical methods. Further, due to the property of AC, it is ideal for systems with high resistance. On the other hand, the maintenance of instruments, electrode structures, and difficulty in data interpretation are examples of its limitations.

#### 2.4.5. MEMS Bioreceptor and Biosensor Recognition Elements

**Figure 15:**
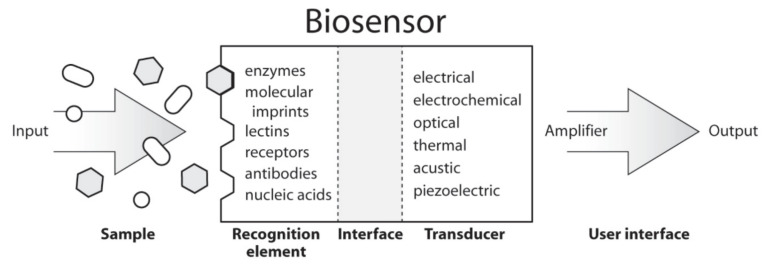
Configuration of a biosensor showing biorecognition, interface, and transduction elements. Reprinted by permission from [110].

Analytical bioreceptor devices that include biologically sensitive elements, which can detect analytes of interest, are comprised of three main elements (a biorecognition element, interface, and transducer). The recognition element is the component of the bioreceptor that is sensitive to the analyte of interest, whereas the transducer transforms biorecognition into a measurable signal. One of these transforming methods is electrochemistry [109]. The most used recognition elements in electrochemical biosensors include antibodies, enzymes, receptors, proteins and peptides, aptamers, nucleic acids, and molecular imprinted polymers, as illustrated in Figure 15 [110].

##### MEMS Antibodies Biosensors (MAB)

**Figure 16:**
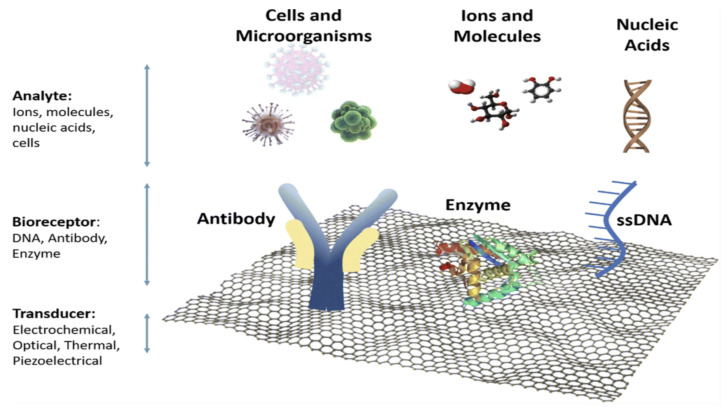
Structure of antibody biosensor. Reprinted by permission from [113].

When the immune system recognizes a non-self entity that has entered the body, it activates a defense mechanism that produces antibodies. Antibodies are one of the main members of the protein family and can attack antigens and lock onto them for removal from the body. There are free sites (paratopes) on the surfaces of antibodies, which can capture antigens. Antigens mostly bind by noncovalent interactions to the antibodies. For most electrochemical sensors, the surface of a biosensor that is modified by antibodies binds and locks with an analyte and provides a signal [81,111,112]. Peña-Bahamonde et al. reported biosensor technologies based on the surfaces of graphene nanomaterials modified with antibodies, enzymes, DNA, and proteins to monitor and determine analytes such as ions, molecules, nucleic acids, and cells, as illustrated in Figure 16 [113]. Du and Zhou used antibody-based biosensor to detect epidemic animal diseases in a pre-clinical phase [114].

##### MEMS Enzyme Biosensors (MEB)

Akin to antibiotics, enzyme biosensors are electrochemical devices that use transducers to capture targets and generate signals. These signals are the result of enzyme reactions with analytes, and transducers can change them to measurable responses (e.g., potential, current, temperature, and catalyzed reactions). Based on the type of transducer in the structure of enzymes, there are three types of methods for the transfer of signals, including potentiometric enzymes, aerometric enzymes, and conductometric enzymes. In most electrochemical biosensors, the surfaces of transducers or matrices are modified by enzymes through physical or chemical methods [115,116]. Nguyen et al. surveyed various methods for the immobilization of enzymes on biosensors for clinical trials [117]. Further, Asal et al. reported advances in enzyme immune-based biosensors for clinical research [118]. Enzyme biosensors exhibit high selectivity. Another advantage of enzymes is that the bioreceptor and transducer are combined into one sensor where, when using this feature, analytes can be detected without reagents. On the other hand, disadvantages include the fact that the lifespans of biosensors are brief due to enzyme binding with surfaces, with the rapid loss of activity [119,120].

##### MEMS Aptamers Biosensors (MApB)

Aptasensors are biosensors that employ aptamers for the detection of analytes. Aptamers are nucleic acids (short single-stranded) and can fold into 3D structures and selectively bind to the target molecules. These materials are produced via an exponential ligand enrichment process [121,122]. Idili et al. used an electrochemical aptamer biosensor to determine phenylalanine in blood [123]. Zhou et al. published a review on the application of altimeter biosensors in cancer research [124]. Aptamers are very selective, are diminutive in size with high stability, which can be easily modified. However, low thermal stability, expense, and low tolerance for pH changes are disadvantages [81,122].

##### MEMS Molecular Imprinted Polymers Biosensors

**Figure 17:**
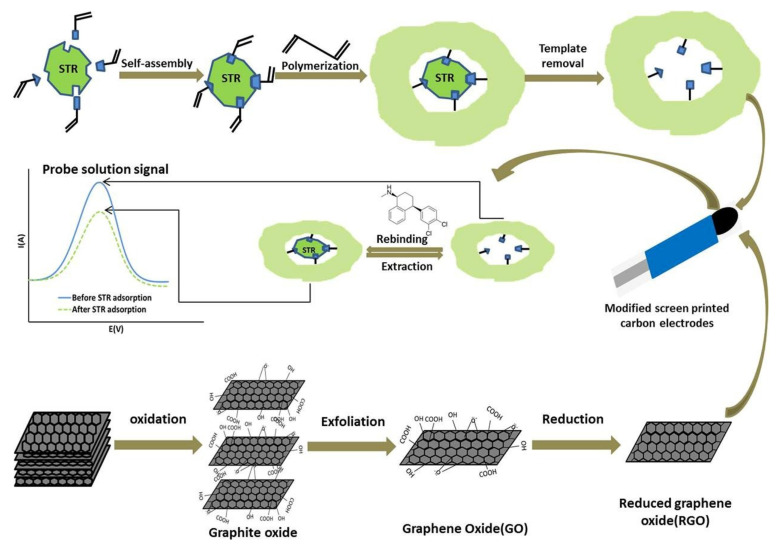
Modification of screen-printed electrode (SPE) with molecular imprinted polymer (MIP). Reprinted by permission from [129]. Copyright 2020 Elsevier.

Molecular Imprinted Polymers (MIPs) are polymers that use imprinting techniques for the selection of molecules. For this method, the template (molecule) initially polymerizes in the presence of monomers and crosslinkers, after which the template is dissolved into a proper solvent and removed from the structure of the polymer, which leaves cavities in the polymer matrix. These cavities are similar to the profile of the original molecular structure, which can then be selectively detected and adsorbed [125]. Feroz, Momina, and Vadgama utilized MIPs to modify electrochemical biosensors and analyze small drugs [126]. Further, Nahhas and Webster used MIPs to improve a detection method for the SARS-CoV-2 virus [127]. Herrera-Chacón et al. developed a MEMS-MIP biosensor based on the modified screen-printed electrode (SPE) [128]. Khosrokhavar et al. employed screen-printed carbon electrode based on molecular imprinted polymers (SPCE-MIP) to determine sertraline antidepressant drug, as illustrated in Figure 17 [129].

MIPs are cost effective; their preparation is relatively easy; and they have high selectivity, high strength, and resistance against high temperatures and pressures. The main disadvantage of this technique is template bleeding, as sometimes many analytes cannot be removed from polymer structure by a solvent, which causes decreasing efficiency and selectivity. Another weakness of this method is the lack of knowledge as to the ratio between template and polymer functions [129,130].

## 3. Summary and Prospects

In recent decades, significant progress has been made on the described analytical techniques. Due to the higher sensitivity and selectivity of chromatographic and electrochemical methods, most spectroscopic and extraction methods have been coupled to improve their performance. Recent advances in electrochemical techniques make them attractive alternatives for the determination of pharmaceutical drugs in clinical trials over chromatography. Advantages such as rapid analysis, non-destructive and inexpensive experimentation, high sensitivity and selectivity, and good data precision and accuracy, were factors that distinguished the electrochemical sensors and biosensors from the other mentioned techniques. Certain issues and disadvantages of chromatographic and spectroscopic methods have been resolved through recently designed MEMS devices. Their performance has been improved via integration with extraction, chromatographic, or spectroscopic techniques. Due to continuously expanding clinical research and pharmaceutical markets coupled with financial and environmental constraints, a bright future can be envisaged for the further development of MEMS sensors and biosensors and their applications in clinical trials.

The challenge with using new electrochemical techniques rather than traditional methods in clinical trials is the disconnect between electrochemical research laboratories and contract research organizations (CROs), their sponsors (pharmaceutical conglomerates), and governmental organisations (FDA or European guidelines). Thus, research laboratories should set their focus on the issues faced by CROs such as time, financial demands, environmental issues, selectivity, and sensitivity, and new electrochemical developments must be directed such that they encompass all CRO requirements. Concurrently, governmental regulatory agencies should keep abreast of research laboratories and provide them with procedural guidelines based on newly developed electrochemical methods for CROs to facilitate and accelerate the deployment of advanced testing for clinical trials. Investing new technologies is essential for CROs; thus, they need to be linked to research laboratories to design innovative techniques based on their requirements. The development and maintenance of this cycle between R&D laboratories, government regulatory agencies, and CROs are critical.

Compared with other methods, electrochemical techniques (particularly MEMS sensors and biosensors) have the potential capacity and necessary properties to be widely applied in clinical trials. Investing the development of electrochemical methods or combining them with other techniques, such as chromatography or spectroscopy, might assist with improving the reliability and accuracy of tests, while addressing the weaknesses of current strategies over the next few decades.

## Figures and Tables

**Table 1:** Application, advantages, and disadvantages of chromatographic methods in clinical trials. and participation rate.

	Method	Separation Basis	Advantages	Disadvantages	Separatable Compounds	Ref.
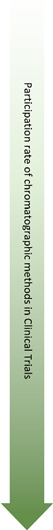	PC	Different rates of separation across paper	Cheap, simple, and user-friendly method	Separate low number of samples, no quantitative analysis	Complex and volatile compounds	[56]
	CC	Size, polarity, or hydrophobicity	Compatible with most complex mixtures, user friendly method	Expensive, time consuming, no quantitative analysis	Nanovesicles	[50]
	GPC	Sieving molecules based on their size	Simple and reliable method	Limitation in volume of sample, low capacity for separation	Proteins, polysaccharides, enzymes, and polymers	[52]
	TLC	Capillary force	Simple, cheap, and user friendly	Limitation for volatile samples and low accuracy and pression	Non-volatile pharmaceutical compounds	[57,58]
	AC	Reversible interaction between analyte and specific ligand	High reliability, great sensitivity and specificity	Complicated maintenance, costly	Purification of enzymes, protein and analyzing vaccines	[53,54,55]
	DLC	Reversible interaction between analyte and specific DYE ligand	Inexpensive and user-friendly	Limitation in selectivity of dye legends	Proteins, enzymes, coenzymes, cofactors, antibodies, and amino acids	[61,62]
	HIC	Interacts with the hydrophobic ligand	High efficiency and sensitivity	Costly buffer (salt)	Separate proteins	[63,64]
	GC	Vaporise samples and sperate through column	Fast and very high efficiency	Only compatible for volatile and thermally stable analytes	Volatile and thermally stable analytes	[59,60]
	IEC	Charge of chemical and biological groups	Efficient and cheap in column price	Costly buffer	Proteins, enzymes, peptides, amino acids, and antibodies	[51]
	RP-HPLC	Pressurising the mixture of mobile phase and sample through the column	Fast, reproducible, and efficient method with high accuracy and precision	Costly and complicated maintenance	Most pharmaceutical compounds	[65,66]

## Data Availability

Not applicable.
